# Phylogenetic Characterization of HIV-1 Sub-Subtype A1 in Karachi, Pakistan

**DOI:** 10.3390/v14102307

**Published:** 2022-10-20

**Authors:** Uroosa Tariq, Jamirah Nazziwa, Sviataslau Sasinovich, Sharaf Ali Shah, Sadaf Naeem, Syed Hani Abidi, Joakim Esbjörnsson

**Affiliations:** 1 Department of Biological and Biomedical Sciences, Aga Khan University, Karachi 74800, Pakistan; 2 Department of Biochemistry, University of Karachi, Karachi 75270, Pakistan; 3 Department of Translational Medicine, Lund University, SE-221 00 Lund, Sweden; 4 Bridge Consultants Foundation, Karachi 75100, Pakistan; 5 Department of Biomedical Sciences, School of Medicine, Nazarbayev University, Astana 010000, Kazakhstan; 6 Nuffield Department of Medicine, University of Oxford, Oxford OX3 7BN, UK

**Keywords:** HIV-1, sub-subtype A1, phylogenetics, phylodynamics, epitopes, *gag*, Pakistan

## Abstract

(1) Background: HIV-1 sub-subtype A1 is common in parts of Africa, Russia, former Soviet Union countries, and Eastern Europe. In Pakistan, sub-subtype A1 is the predominant HIV-1 subtype. Preliminary evidence suggests that distinct strains of HIV-1 sub-subtype A1 are circulating in Pakistan; however, an in-depth molecular phylogenetic characterization of HIV-1 sub-subtype A1 strains in Pakistan have not been presented. We performed a detailed characterization of the HIV-1 sub-subtype A1 epidemic in Pakistan using state-of-the-art molecular epidemiology and phylodynamics. (2) Methods: A total of 143 HIV-1 sub-subtype A1 *gag* sequences, including 61 sequences generated specifically for this study from PLHIVs part of our cohort, representing all sub-subtype A1 *gag* sequences from Pakistan, were analyzed. Maximum-likelihood phylogenetic cluster analysis was used to determine the relationship between Pakistani sub-subtype A1 strains and pandemic sub-subtype A1 strains. Furthermore, we used signature variation, charge distribution, selection pressures, and epitope prediction analyses to characterize variations unique to Pakistani HIV-1 strains and establish the association between signature variations and Gag epitope profile. (3) Results: The HIV-1 sub-subtype A1 sequences from Pakistan formed three main clusters: two that clustered with Kenyan sequences (7 and 10 sequences, respectively) and one that formed a Pakistan-specific cluster of 123 sequences that were much less related to other sub-subtype A1 sequences available in the database. The sequences in the Pakistan-specific cluster and the Kenyan reference strains exhibited several signature variations, especially at amino acid positions 312, 319, 331, 372, 373, 383, and 402. Structural protein modeling suggested that amino acid changes in these positions result in alterations of the Gag protein structure as well as in Gag-specific T-cell epitopes. (4) Conclusions: Our results suggest that the majority of the Pakistan HIV-1 sub-subtype A1 strains were unique to Pakistan and with a specific mutation pattern in Gag.

## 1. Introduction

Human immunodeficiency virus type 1 (HIV-1) is a major global health concern and is responsible for approximately 37.6 million infected individuals worldwide [1]. A high level of genetic diversity is a characteristic feature of HIV-1, attributed to error-prone replication, recombination between HIV-1 strains, and evolution under immune selection pressure [2,3]. Over time, HIV-1 has diversified into several hundred lineages (called subtypes, sub-subtypes, or circulating or unique recombinant forms [CRFs or URFs]), often specific to a geographic region, e.g., sub-subtype A1 in East Africa [4,5] and East Europe [6,7], sub-subtype A3 in West Africa [8,9,10], region-specific strains of subtype B in Thailand, Brazil, Korea, Trinidad, and Tobago [11], and HIV-1 subtype C in Brazil [12].

HIV-1 tends to evolve under selection pressure exerted by the host immune system, where human leukocyte antigens (HLAs) play a major role in the CD8+ T-cell immunity [13]. HLA diversity worldwide is shaped by demographics and natural selection [14], and the HLA region on chromosome 6 is a highly polymorphic region of the human genome with >7000 HLA alleles [15]. Moreover, the HLA frequency can differ at the population level and result in differential inter-population selection pressure which may affect the evolution of HIV-1 in a population-specific manner [16].

The HIV-1 epidemic in Pakistan has been expanding rapidly and a large increase (57%) in new HIV-1 infections has been observed during the last ten years [17,18]. These infections have now bridged from high-risk groups, such as people who inject drugs (PWID) and men who have sex with men (MSM), into the low-risk communities, including women and children [19,20]. The HIV-1 epidemic in Pakistan is mainly driven by HIV-1 sub-subtype A1. However, other HIV-1 strains have been found in different outbreaks in the country [19,20]. Several of them have emerged as recombinant forms including sub-subtype A1, e.g., CRF02/A1 [21], indicating an increasing diversity in circulating HIV-1 strains in Pakistan. However, an in-depth molecular characterization of HIV-1 sub-subtype A strains in Pakistan has not been presented. Moreover, the relationship between Pakistani sub-subtype A1 strains and other sub-subtype A1 strains is largely unknown. In the current study, we analyzed HIV-1 *gag* sequences to characterize the HIV-1 sub-subtype A1 epidemic in Pakistan by state-of-the-art molecular epidemiology and phylodynamics.

## 2. Materials and Methods

### 2.1. Study Population

In total, 143 HIV-1 *gag* sequences were analyzed. Of these, 61 were generated from whole blood samples (3–5 mL whole blood from each study participant) collected from people living with HIV-1 (PLHIV) enrolled in a Pakistan high-risk community HIV-1 cohort in Karachi-Pakistan. The age of the participants ranged from 18 to 64 years, and most of them were male (92%), with 64% being on antiretroviral therapy (ART, Table 1). Recorded information on risk group included people who inject drugs (PWID, 43%), men who have sex with men (MSM, 13%), spouse of an HIV positive individual (SP, 2%), and heterosexual (5%, Table 1). The majority of participants were diagnosed for HIV-1 infection between 2011 and 2015 (61%), and all samples were collected in 2015 (Table 1, [3]). Samples from PLHIV were recruited after obtaining written informed consent from each participant. Basic information regarding age, sex, ethnicity, ART usage, and high-risk behavior was obtained from each participant. Ethical approval for the study was obtained from the Ethical Review Committees of Aga Khan University (4189-BBS-ERC-16). In addition to the new sequences generated from these samples (as described below), 82 HIV-1 sub-subtype A1 *gag* sequences from Pakistan (previously deposited to the Los Alamos Database [https://www.hiv.lanl.gov]; accessed on 1 October 2018) were included in the study. All accession numbers are listed in Supplementary Table S1.

### 2.2. HIV-1 gag Gene Amplification and Sequencing

DNA extraction was performed by the QIAamp DNA Blood Mini Kit from Qiagen (Hilden, Germany), according to the manufacturer’s instructions. The HIV-1 *gag* gene was amplified by a two-step nested PCR strategy, using three different approaches for the first round. The first round primers in the first, second, and third approaches were: GOPF (5′-CTCTCGACGCAGGACTCGGCTTGC-3′, HXB2 [accession number: K03455] positions 683–706), GOPR (5′-CCAATTCCCCCTATCATTTTTGG-3′, 2382–2404); NOPF1 (5′-CAAAGATCTCTCGACGCAG-3′, 676–694), NOPR1 (5′- CTGTATCATCTGCTCCTGTG-3′, 2327–2346); and OPPF (5′-CTAGCAGTGGCGCCCGAACA-3′, 629–648) and OPPR (5′- CTAATACTGTATCATCTGCTCCTGT-3′, 2328–2352). The primers GIPF (5′-GAGGCTAGAAGGAGAGAGATGGG-3′, 772–794) and GIPR (5′-TTATTGTGACGAGGGGTCGTTGCC-3′, 2269–2292) were used for nested amplification. The reaction mixture of 25 μL for both first and second-round PCR contained 5 μL of PCR buffer (5 × Green GoTaq^®^ Flexi Buffer, pH 8.5), 2 mM MgCl_2_, 400 μM dNTPs, 0.3 U GoTaq Polymerase (Promega M3001, Madison, WI, USA), and 0.48 pmol primers. The thermocycling conditions were as follows: denaturation at 95 °C for 5 min, followed by 35 cycles of denaturation at 95 °C for 1 min, annealing at 58 °C (first approach)/50 °C (second approach)/48 °C (third approach) for 1 min, and extension at 72 °C for 1 min, with a final extension of at 72 °C for 15 min. One μL of the first-round PCR product was used for the second-round PCR. The thermocycling conditions were as follows: Denaturation at 95 °C for 5 min, followed by 35 cycles of denaturation at 95 °C for 1 min, annealing at 60 °C for 1 min, and extension at 72 °C for 1 min, with a final extension of at 72 °C for 15 min. The amplified products were electrophoresed on a 1.2% agarose gel (Sigma-Aldrich A1296; St. Louis, MO, USA), stained by ethidium bromide, and visualized under ultraviolet light. PCR products were sequenced by Macrogen Inc., Korea (Supplementary Table S2).

### 2.3. Subtyping Determination

For subtype determination, *gag* sequences from Pakistan were aligned with the most recent (2010) HIV-1 subtype reference sequence dataset containing all group M sequences and circulating recombinant forms (CRFs, https://www.hiv.lanl.gov/; accessed on 1 October 2018) using the Clustal algorithm, followed by manual editing in Geneious v8.1.9 [22]. The *gag* alignment was submitted for maximum likelihood (ML) phylogenetic reconstruction in PhyML 3.0 using the following parameters: general time-reversible (GTR) model of nucleotide substitution with a gamma-distributed rate heterogeneity. Branch support was assessed by an approximate likelihood-ratio test based on the Shimodaira–Hasegawa-like procedure (aLRT-SH) [23]. The inferred tree was assessed and annotated in FigTree (v1.4.3; http://tree.bio.ed.ac.uk/software/figtree/; accessed on 10 October 2018). Nodes with aLRT-SH support ≥0.9 were considered significant.

### 2.4. Cluster Analysis

A BLAST approach was used to generate a reference sequence dataset from NCBI Genbank [24] based on similarity with the Pakistani HIV-1 sequences, as previously described [25]. Briefly, the 10 closest matches for each Pakistani sequence were selected, after which duplicate hits were removed using the program skipredundant.exe (threshold 98%) from the EMBOSS package [26]. Information about the collection date, sampling country, and route of transmission was also collected for each sequence. Subsequently, the sequence alignment was used to determine a maximum likelihood (ML) tree as described above.

### 2.5. Time-Scaled Phylogenetic Reconstruction

Time-scaled phylogenetic reconstruction was done using the Bayesian Markov chain Monte Carlo (MCMC) method as implemented in BEAST package v1.10.2 [27] with the following settings: HKY + G nucleotide substitution model with codon partitioning; relaxed clock with an uncorrelated lognormal rate distribution; and SkyGrid demographic model [28]. The prior ulcd. mean was set to uniform with an initial 0.001 (upper limit 1, and lower limit 1.0 × 10^−6^). The posterior probability ≥0.99 was used to determine monophyletic clusters. MCMC chains were run for 2 × 10^8^ generations and sampled every 20,000 steps. Maximum clade credibility (MCC) trees were generated in TreeAnnotator v1.10.2 [29] after 10% burn-in. Time trees were labeled in FigTree v1.4.4 [30]. Two individual runs were performed for each dataset, with log files combined in LogCombiner V1.10.2 [31].

### 2.6. Signature Variation(s) Analysis and Charge Distribution

Consensus nucleotide sequences were generated in Geneious v8.1.9 [22], followed by translation to corresponding amino acid sequences in ExPASy [32] for protein-based analysis. To identify the molecular level variations behind the divergence and temporal changes, the signature variations (defined as variations unique to a particular set of sequences) were identified using the viral epidemiology signature pattern analysis (VESPA) tool available in the Los Alamos HIV Database [33]. The statistical significance of the ratio of the identified unique sites between the strains was determined using the Chi-square test using GraphPad (https://www.graphpad.com/quickcalcs/chisquared1/; accessed on 1 January 2019). Amino acid variations were plotted using the WebLogo tool [34]. The effects of signature variations on the biophysical properties of the sequences were determined by calculating the differences in charge distribution on each amino acid along the length of the HIV-1 sub-subtype A1 Gag protein. Charged amino acids (arginine, lysine, glutamic acid, aspartic acid, and histidine) and potential N-linked glycosylation sites (PNGSs) were determined using an in-house Perl script based on the rules set in GLYCOSITE, as described previously [2]. The amino acid positions in HIV-1 Gag were determined using HXB2 (accession number K03455) as a reference. The statistical significance of the net charge and total charge between strains was determined using an unpaired t-test employed in Prism 9 version 9.3.1 (GraphPad Software, San Diego, CA, USA).

### 2.7. Renaissance Counting

To determine sites under selection, ratios of non-synonymous (dN) and synonymous (dS) substitutions were estimated by renaissance counting, as implemented in BEAST v1.10.2 using the HKY85 nucleotide substitution model, three-site codon partitioning, and an uncorrelated relaxed molecular clock with lognormal distribution [2,35]. The MCMC chain length was set at 2 × 10^8^.

### 2.8. Epitope Mapping

To determine the effect of significant variations in cytotoxic T-cell (CTL) and helper T-cell epitopes, pre-defined CTL and helper T-cell epitopes were retrieved from the HIV molecular immunology database (www.hiv.lanl.gov/content/immunology/index). For comparison, epitopes were then mapped on the consensus Gag protein sequences of the Pakistani and reference datasets, respectively. Furthermore, HLA anchoring residues and restricting HLA alleles for each epitope were predicted from consensus sequences using the Motif Scan tool available in the Los Alamos Database [36].

### 2.9. Protein Structure Modeling

Finally, the effect of signature variations on protein structure was determined using protein homology modeling, and secondary and tertiary structures were modeled using the ab-initio protein structure prediction tool QUARK (https://zhanglab.ccmb.med.umich.edu/QUARK, accessed on 1 January 2019) [37]. The similarity between the generated tertiary structure models was determined by superimposing the two protein models using the Click server (http://cospi.iiserpune.ac.in/click/, accessed on 1 January 2019) [38]. Finally, sites with signature variations were marked in Discovery Studio Visualizer V17.2.0 [39].

## 3. Results

### 3.1. Subtyping and Cluster Analysis of HIV-1 Sub-Subtype A1 in Pakistan

**Figure 1:**
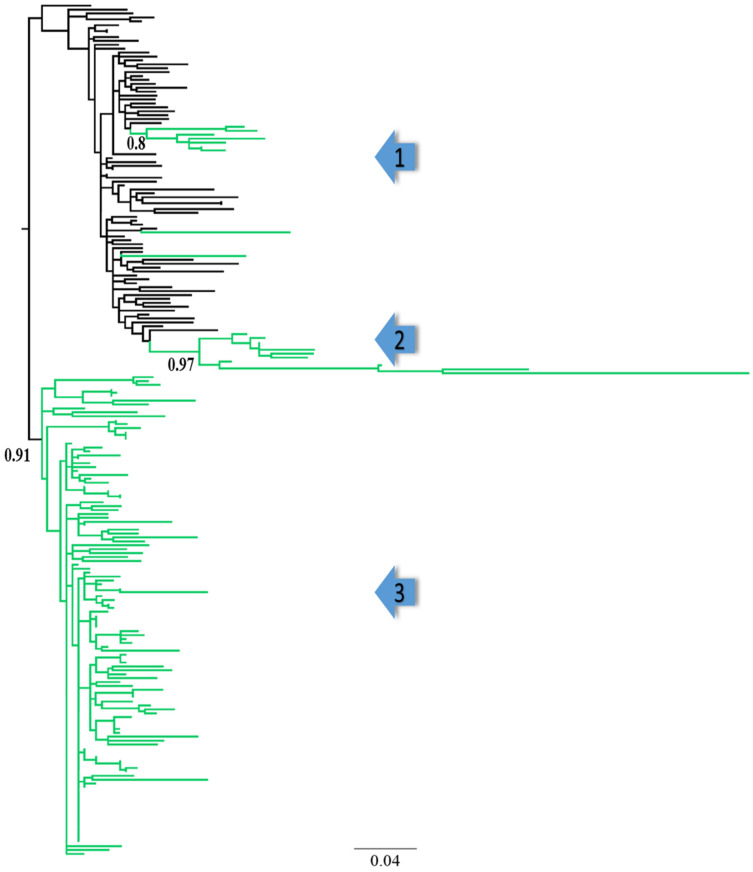
Phylogenetic clusters of HIV-1 sub-subtype A1 in Pakistan. Tips in green and black represent, respectively, Pakistani and Kenyan (reference) HIV-1 sub-subtype A1 sequences. Arrows indicate clusters 1, 2, and 3 in the phylogenetic tree, while the aLRT-SH support values of the three clusters are also shown.

The subtype analysis indicated that all 143 *gag* sequences from Pakistan were sub-subtype A1 (Supplementary Figure S1). After the removal of duplicate sequences (from the total number of reference sequences obtained from the database), the dataset was reduced to 80 patient-unique reference sequences from Genbank for cluster analysis [24]. Except for two sequences, all Pakistan sequences clustered together in three Pakistani-specific clusters (Clusters 1–3, Figure 1), whereof most sequences were found in one large Pakistani-exclusive cluster (Cluster 3, n = 123 sequences, Figure 1). The remaining Pakistani sequences formed two separate clusters with sequences from Kenya (Cluster 1 and Cluster 2, Figure 1). To assess if the Kenyan reference sequences were representative of the main HIV-1 sub-subtype A1 epidemic in Kenya, we reconstructed a separate ML tree based on all available Kenyan sub-subtype A1 sequences (n = 2045) in the Los Alamos Sequence Database (accession numbers in Supplementary Table S3). The analysis showed that the reference sequences were intermingled with the remaining Kenyan sequences, indicating that the reference sequences were representative of the main HIV-1 sub-subtype A1 epidemic in Kenya (Supplementary Figure S2). Due to their close genetic relationship with the Pakistani sequences, these Kenyan sequences were used as references in subsequent comparative analyses.

### 3.2. Date of Origin and Evolutionary Dynamics of HIV-1 Sub-Subtype A1 in Pakistan

**Figure 2:**
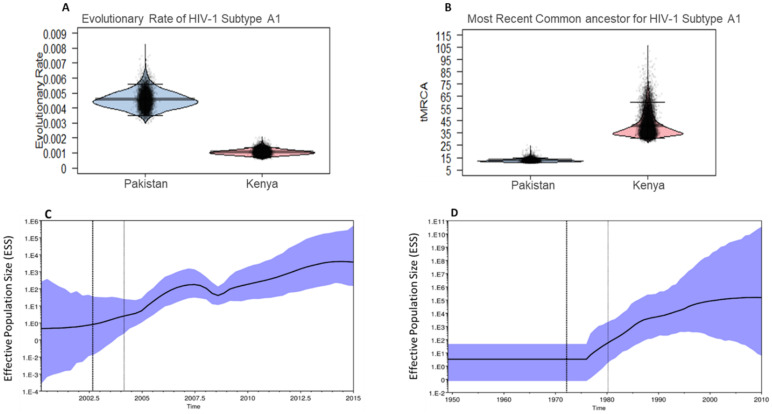
Demographic History of HIV-1 sub-subtype A1 in Pakistan. (**A**) The pirate plot of evolutionary rate. (**B**) Time to the most recent common ancestor (tMRCA) of the Pakistani and Kenyan HIV-1 sub-subtype A1 strains. (**C**,**D**) Bayesian Skygrid plots of the number of effective infections over time for the Pakistani (**C**) and Kenyan (**D**) HIV-1 sub-subtype A1 strains. The dotted lines represent the median tMRCA of the strain. The blue area (C and D) represents a lower and upper 95% higher posterior density (HPD).

To further characterize the HIV-1 sub-subtype A1 epidemic in Pakistan, we performed an in-depth phylodynamic analysis of the main Pakistani cluster, Cluster 3 (n = 123). For comparison and to gain further insight, we also analyzed the Kenyan HIV-1 sub-subtype A1 cluster (n = 51, excluding the 19 Pakistani sequences, Figure 1). Some sequences from the Kenyan reference dataset were removed because of poor temporal signal or possible recombination (based on clustering pattern). The median time to the most recent common ancestors (tMRCA) was 2002 (95% highest posterior density (HPD): 2000–2004) for the Pakistani sub-subtype A1 cluster 3, and 1969 (95% HPD: 1949–1980) for the Kenyan cluster (Figure 2A). The mean evolutionary rate for the Pakistani cluster 3 was 4.2 × 10^−3^ substitution/site/year (s/s/y, 95% HPD interval: 3.5 × 10^−3^–5.0 × 10^−3^), compared with 1.0 × 10^−3^ (95% HPD Interval: 7.1 × 10^−4^–1.3 × 10^−3^) for the Kenyan cluster (Figure 2B).

Skygrid analysis indicated that the effective number of HIV-1 sub-subtype A1 infections increased in Pakistan from 2002 to 2007 (Figure 2C). A potential drop in effective infections was indicated at around 2008, prior to a modest continuous increase between 2009 and 2015. The analysis of the Kenyan cluster indicated a sharp increase in effective infections between 1975 and 1995, before stabilizing after 2000 (Figure 2D). However, the apparent stabilization after 2000 was accompanied by a large variance and should be interpreted with caution.

### 3.3. Analysis of Molecular Properties between Pakistani and Kenyan HIV-1 Sub-Subtype A1 Sequences

**Figure 3:**
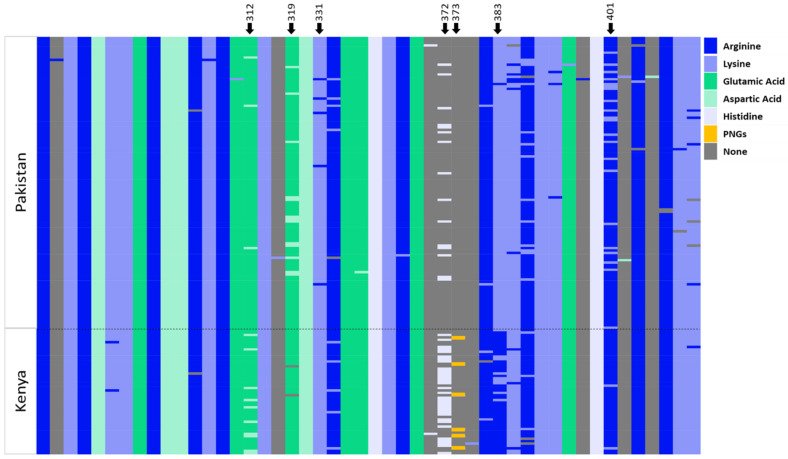
Biophysical properties of amino acids on sites of Kenyan and Pakistani HIV-1 sub-subtype A1 strain. Blue and light blue colors represent arginine and lysine amino acids, whereas the green and light green represent glutamic acid and aspartic acid, respectively. The black arrow represents sites with visible differences between the two strains.

The VESPA analysis indicated seven amino acid sites that differed between Pakistani and Kenyan HIV-1 sub-subtype A1 sequences (Table 2). The total and net charges of HIV-1 sub-subtype A1 sequences from Pakistan and Kenya were not significantly different (*p* = 0.886) (Supplementary Table S4). However, differences in charged amino acids were observed at positions 312, 319, 331, 372, 373, 383, and 402 with reference to the HXB2 position (Figure 3, Supplementary Table S6). One PNGS (at position 373) was only found among the Kenyan sequences (Figure 3, Supplementary Table S5). Moreover, the negatively charged glutamic acids found at positions 312 and 319 among the Kenyan sequences were replaced by a negatively charged aspartic acid among the Pakistani sequences (Figure 3, Supplementary Table S6). Furthermore, the neutral amino acid histidine at position 372 was observed more frequently among the Kenyan sequences (n = 33, 65%) as compared to the Pakistani sequences (n = 15, 12%, *p* < 0.001, two-tailed Fisher’s exact test, Figure 3, Supplementary Table S6). Similarly, position 383 of Gag also exhibited a change in amino acid charge, where arginine was completely replaced with the positively charged amino acid lysine in Pakistani sequences (Figure 3, Supplementary Table S6).

### 3.4. Gag Sites under Selection in Pakistani and Kenyan HIV-1 Sub-Subtype A1 Strains

**Figure 4:**
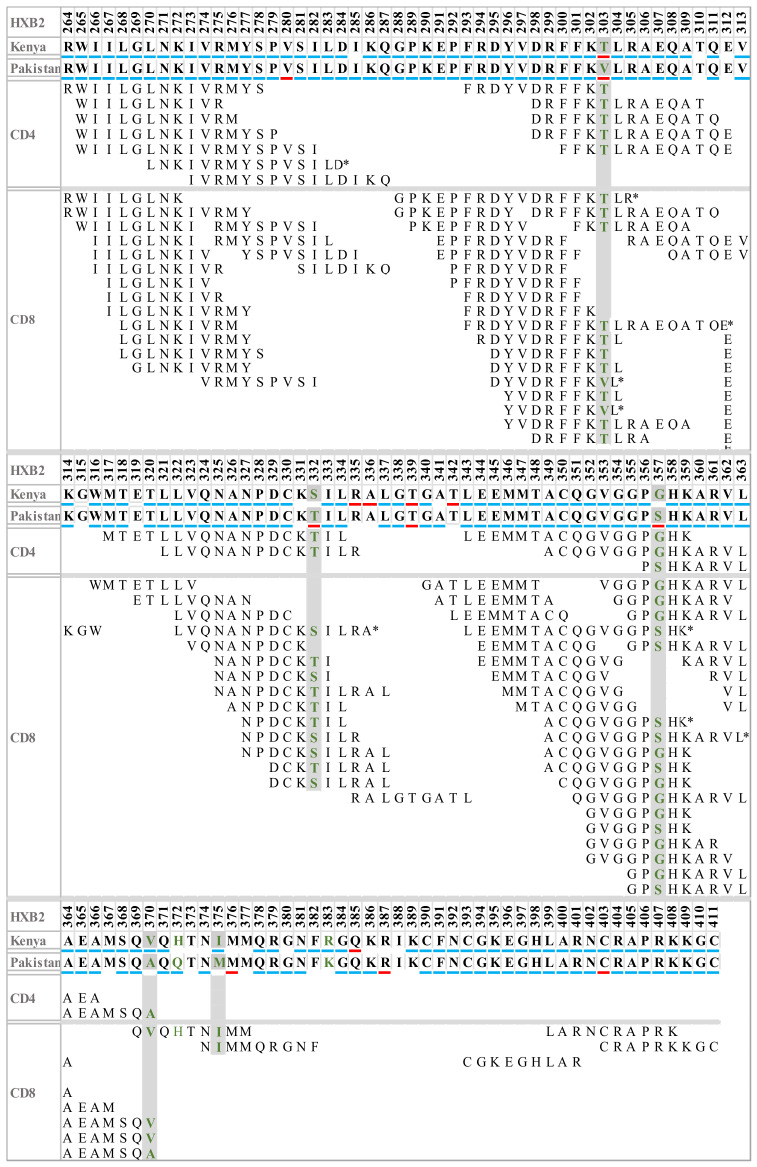
CD4 and CD8 T-cell epitope map on HIV-1 sub-subtype A1 Gag. The amino acids under negative and positive selection pressure are underlined with blue and red colors, respectively. Neutral sites are not underlined. Amino acids dissimilar between the Pakistani and Kenyan sequences are represented with the green color font. The grey color line separates the CD4 and CD8 T-cell epitopes and the steric represents the epitope variant.

The ratio of non-synonymous (dN) and synonymous (dS) substitution was determined to assess site-specific selection in Pakistani and Kenyan HIV-1 sub-subtype A1 Gag sequences. The analysis showed that eight sites in the Pakistani sequences and six sites in the Kenyan sequences were under significant positive selection pressure (Figure 4). Moreover, the HIV-1 Gag positions 303 and 339 (identified as signature sites distinguishing Pakistani and Kenyan sequences) were under positive selection in both sequence sets, whereas positions 332 and 357 were under positive selection in Pakistani sequences only (Figure 4).

### 3.5. Sequence Variation in HIV-1 Gag T-Cell Epitopes

In the next step, we evaluated the effects of mutations (the amino acids dissimilar between Pakistani and Kenyan sequences (303, 332, 357, 370, 372, 375, and 383)) on CTL and helper T-cell epitope generation (Figure 4). The number of identified epitopes between Kenyan (CD4^+^ = 7 and CD8^+^ = 27) and Pakistani (CD4^+^ = 4 and CD8^+^ = 15) consensus sequences differed due to amino acid polymorphisms in epitope regions. For example, the presence of threonine (T) at amino acid position 303 in the Kenyan sequence matched five CD4^+^ and ten CD8^+^ T-cell epitopes, respectively (Figure 4). In contrast, a valine (V) in this position in the Pakistani sequence only matched two CD8^+^ T-cell epitopes (Figure 4). In contrast, some signature variations resulted in the prediction of CD4^+^ T-cell epitopes in Pakistani sequences only, such as sites 332 and 370, which matched two and one CD4^+^ T-cell epitopes, respectively (Figure 4). Moreover, amino acid site 375 in the Pakistani strain was represented by two CD8+ T-cell epitopes, whereas the same site in the Kenyan strain did not match any previously described epitopes (Figure 4).

### 3.6. Sequence Variation in HLA Binding Motifs and Epitopes

Next, we evaluated the effects of mutations (the amino acids dissimilar between Pakistani and Kenyan sequences (303, 332, 357, 370, 372, 375, and 383)) on HLA anchorage. In total, 176 epitopes and 20 unique HLA anchoring residues were found in the Pakistani consensus sequence, compared with 175 epitopes and 19 residues in the Kenyan consensus sequence (Table 3 and Supplementary Table S5). The sub-subtype A1 signature sites 370, 372, and 375 in the Pakistani sequence (containing amino acids A, Q, and M, respectively) were associated with 7 unique CD4^+^/CD8^+^ T-cell epitopes, whereas the corresponding sites in the Kenyan sequence (containing amino acids V, H, and I, respectively) were associated with 11 CD4^+^/CD8^+^ T-cell epitopes (Table 3 and Supplementary Table S5). An alanine at position 370 in the Pakistani sequence resulted in three unique CD4^+^/CD8^+^ T-cell epitopes, in contrast to the Kenyan sequence, where a valine at the same position resulted in five unique CD4^+^/CD8^+^ T-cell epitopes (Table 3 and Supplementary Table S5).

The signature position 303 in the Pakistani sequence (containing a valine) was identified as an anchor site in nine unique epitopes restricted by the following type I HLAs: A*0206, B*3501, B*5103, Cw*0601, and Cw*0602; and type II HLAs: DPA1*0102, DPA1*0201, DPB1*0201, DPB1*0401, DRB1*0401, DRB1*0901, and DRB4*0101; whereas a threonine at position 303 in the Kenyan sequence was associated with only one unique epitope of four amino acids (FFKT) with anchor residues in the following type II HLAs: DRB1*0301 and DRB3*0201 (Table 3). Moreover, site 332 in the Pakistani sequence containing a threonine was not associated with any epitope, whereas the presence of a serine at the same site in the Kenyan dataset was associated with the epitope NANPDCKSI that is restricted by HLA B*7801 (Table 3).

### 3.7. Structural Diversity

**Figure 5:**
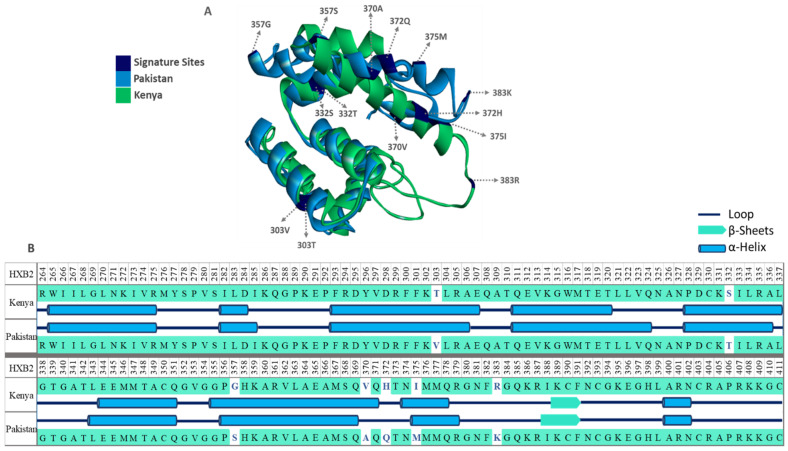
Structural diversity of HIV-1 sub-subtype A1 Gag protein in Kenya and Pakistan. (**A**) Superimposed Gag structure from Pakistan (Blue) and Kenya (Green) with signature sites highlighted in dark blue color. (**B**) Predicted Secondary structure (α-helix = Blue cylinder, β-sheets = teal arrow, and loop = dark blue line) mapped on Gag from both strains.

The structure of the HIV-1 Gag protein was predicted using the ab initio method in the QUARK tool [37] to assess the potential effects of differences in the identified amino acid differences between the Kenyan and Pakistani consensus sequences on the Gag protein structure. The analysis suggested several secondary and tertiary structural differences (Figure 5). For example, differences in positions 303, 332, 357, 370, and 372 resulted in a longer α-helix in the Kenyan sequence as compared to the Pakistani Gag protein sequence (Figure 5A,B). Similarly, the signature position 375 in the Kenyan sequence changed the start position of α-helix in Gag protein (start-end: 374–378, with reference to HXB2 position) as compared to the Pakistani sequence (start-end: 375–379, with reference to HXB2 position, Figure 5A,B). Additionally, the signature pattern at position 383 elongated the β-sheet downstream in Gag from the Pakistani HIV-1 strain compared to the Kenyan strain (Figure 5A,B). The superimposition of tertiary structures of Gag from the Kenyan and Pakistani strains indicated a 64% similarity between the two structures. The α-helix extension caused by differences in sites 303, 332, 357, 370, and 372, in the Kenyan strain, altered the overall folding of the protein and affected the complete superimposition of the protein. The variation at position 383 in the Kenyan strain resulted in a bigger and more exposed loop compared to the Pakistani strain (Figure 5A,B).

## 4. Discussion

In this study, we characterized the HIV-1 sub-subtype A1 epidemic in Pakistan on the basis of the phylogenetic and molecular properties of *gag*. Our dataset includes all known HIV-1 sub-subtype A1 *gag* sequences. The analysis showed that the HIV-1 sub-subtype A1 sequences from Pakistan formed three main clusters, whereof two clustered with Kenyan sequences suggesting a close relationship between the Kenyan and the Pakistani HIV-1 sub-subtype A1 epidemics. This is in line with a previous study on pan-epidemic strains of sub-subtype A1 (performed on sub-subtype A1 sequences collected up to 2010) [40]. However, the third and larger cluster was much less related to other available sub-subtype A1 sequences, also suggesting the presence of a Pakistani-specific HIV-1 sub-subtype A1 strain. To the best of our knowledge, this Pakistani-specific strain has not been described before, and this may reflect an evolving and distinct HIV-1 epidemic in Pakistan. The phylodynamic analysis indicated that this strain emerged around 2002 (95% HPD: 2000–2004) and had an approximately four times higher evolutionary rate compared with the Kenyan strain. This could be due to the selection of evolutionary ‘fit’ and transmissible strain, capable of spreading rapidly to high-risk groups such as PWID and MSM [41,42,43]; however, this could not be ascertained due to a lack of availability of precise sequences and epidemiological information from that period.

Moreover, the evolutionary rate of the Pakistani strain was approximately twice as high compared with the HIV-1 *gag* evolutionary rate in the global sub-subtype A1 epidemic (median rate: 2.3 × 10^−3^ s/s/y), as recently determined by Patino-Galindo et al. [44]. This may reflect the recent emergence of a Pakistani-specific HIV-1 strain that still has not fully adapted to the Pakistani population. Indeed, the evolution of population-specific strains is not an unusual phenomenon in HIV-1 infection, and several studies have reported on different subtypes that have evolved into population-specific sub-strains (e.g., the Thai subtype B, Brazilian subtype B, Korean subtype B, Trinidad and Tobagonian subtype B, and Ethiopian and East African subtype C strains) [11,45]. Furthermore, the phylodynamic analysis of the Pakistan-specific lineage suggested a rapid increase in the number of effective infections and transmissions following the emergence of the strain in 2002. This is in agreement with the HIV-1 surveillance data for Pakistan, showing a rapid increase in new cases in the country, especially during the last 10 years [1,19,41,46].

Next, we compared the consensus sequences of the Pakistani and the Kenyan HIV-1 sub-subtype A1 strains for the presence of mutations and variations unique to Pakistani or Kenyan datasets (referred to as signature sites). Interestingly, some of the identified sites (303, 332, 370, 372, and 375) have previously been shown to be specific for sub-subtype A1, and even specific to Kenyan and Pakistani sequences [16]. However, in contrast to the previous study, which was focused on only Pakistani and Kenyan patient-derived sequences, this study included all available sub-subtype A1 *gag* sequences deposited from Pakistan (patient-derived and database sequences), which not only confirms the previous findings but also identifies additional sites, such as position 373, to be uniquely different in Kenyan sequence, as compared to Pakistani sequences, because of the presence of PNGSs. Glycosylation is known to affect the function of Gag protein in HIV-1; for example, previous studies have demonstrated that the glycosylated Gag protein mimics the Nef function in *nef* deficient HIV-1 by restoring and rescuing its infectivity [47]. In addition to glycosylation, changes in the net charge can affect protein stability. For example, in GFP protein, instead of lysine, the presence of arginine, which has three nitrogen atoms and is capable of forming hydrogen bonds with neighboring amino acids (Pro75 and Asp76), leads to protein stability [48]. Interestingly, at signature position 383 in NC (nucleocapsid region), arginine was found in the Kenyan strain, which was replaced with lysine in the Pakistani strain. Our observations cannot ascertain the link between the transmission advantage of the Kenyan compared to the Pakistan strain. However, further studies to investigate the role of amino acid charge differences in Gag structure and functions can provide further insights into how the difference in the charge of Gag amino acids can affect viral infection dynamics.

HIV-1 Gag is relatively conserved compared with other HIV-1 proteins and contains several immunodominant regions [49]. Mutations in the *gag* gene have previously been associated with increased tolerance against drugs or immune activity. For example, in vitro studies have demonstrated that mutations in the capsid can produce negative effects on virus infectivity [50]. Moreover, mutations in the Gag p2/NC (spacer peptide 1/nucleocapsid) cleavage site can reduce the efficacy of protease inhibitors, which ultimately leads to treatment failure and propagation of the infection [51]. Other effects include changes in epitope sequence, which may abrogate the recognition of epitopes, particularly T-cell epitopes, leading to immune escape [40,52]. An in-depth molecular analysis of Gag indicated signature amino acid variation in Pakistani HIV-1 sub-subtype A1, which may have evolved under immune pressure. The presence of valine at signature position 303 of Gag resulted in the generation of more epitopes, as valine has been predicted to serve as an anchoring residue for five HLA type I and seven HLA type II molecules [36]. On the contrary, the presence of threonine at the same position has been associated with a decreased number of epitopes [16]. In addition, a recent study on HIV-1 subtype C showed an association between the selection pressure exerted by HLA Cw*0303/Cw*0304 and amino acid variation at position 303 in the HLA-restricted HIV-1 Gag epitope YVDRFFK**T**L [53]. Moreover, the substitution of wild-type amino acid T with A/I/V at position 303 in Gag has been suggested to reduce the CD8+ T-cell recognition of the epitope [53]. Similarly, the mutation T303A/I has been shown to decrease HIV-1 infectivity rates, whereas HIV-1 with T303V showed similar replicative fitness compared with the wild-type strain [53]. In contrast, the signature position 357, with a serine in the Pakistani strain, was found to reduce the number of CD4^+^/CD8^+^ T-cell epitopes compared with the Kenyan strain that had a glycine in this position. This observation is supported by a previous study indicating that the set of four substitutions (79F+228L+286K+357G) can reduce the replicative capacity of NL4-3 (mutant) by 2-fold [54]. However, due to limited in vitro and in vivo data on epitopes specific for HIV-1 sub-subtype A1, a detailed assessment of the relationship between signature mutations and immune escape cannot be established and warrants further in vitro studies to confirm this phenomenon. However, it is important to mention that since more than half of the patients were on ART, and archival HIV-1 DNA genomes were amplified, it may be difficult to assess actual immune escape in Gag in these patients.

The HIV-1 inhibitor Bevirimat (BVM) binds to the Gag spacer regions and blocks the cleavage of the protein [55]. The amino acid positions 370, 372, and 375 found to be different between the Pakistani and Kenyan strains are the known binding residues of Gag inhibitors [55]. In this study, we found a signature variation at position 370 of the Gag protein (alanine and valine in the Kenyan and Pakistani strains). Interestingly, the V370A polymorphism has previously been shown to reduce the BVM susceptibility by 40-fold. Although this has to be verified by in vitro experiments, this opens up the possibility that the Pakistani HIV-1 sub-subtype A1 strain may be resistant to BVM [55].

## 5. Conclusions

In summary, this study increases our understanding of the HIV-1 sub-subtype A1 introduction, evolution, and diversification in Pakistan. The results suggest that HIV-1 sub-subtype A1 strains are unique to Pakistan with phylogenetic linkages to the Kenyan strains. Additionally, Pakistani sub-subtype A1 strains have accumulated certain unique mutations in the *gag* gene that may facilitate HIV-1 adaptation to host selection pressures and more effective transmission of the virus to different at-risk groups. Further studies are needed to fully disentangle the role of the identified mutations in virus transmission dynamics.

## Figures and Tables

**Table 1:** Characteristics of study participants.

Category/Variable	Total No. (%)
Age (years)	
15–24	11(18)
25–64	50 (82)
Sex	
Male	56 (92)
Female	5 (8.12%)
Marital Status	
Married	31 (51)
Single	25 (41)
Not declared	5 (8)
ART History	
Experienced	39 (64)
Naïve	19 (31)
Not declared	3 (5)
Risk group	
PWID	26 (43)
MSM	8 (13)
HET	3 (5)
SP	5 (2)
NA	2 (3)
Year of Diagnosis	
2000–2005	8 (13)
2006–2010	5 (8)
2011–2015	37 (61)
Unknown	11 (18)
Date of sampling	2015

Abbreviations: MSM: men who have sex with men; PWID: people who inject drugs; HET: heterosexual; SP: spouse of an HIV positive individual; NA: not available.

**Table 2:** **Signature Variation Pattern of HIV-1 Sub-Subtype A1 in Pakistan in comparison with the Kenyan reference dataset.** The predominant amino acid variant in the Pakistani and Kenyan reference dataset is represented in 1 and 4 rows of the table, respectively. The frequencies of each amino acid variant are mentioned below the variant, whereas the highest frequencies of variants in the Pakistani and Kenyan reference dataset are shown in bold. The position of the amino acids concerning the HXB2 is mentioned in the last row.

Pakistan	V	T	S	A	Q	M	K
Frequency in Pakistan	**0.854**	**0.911**	**0.797**	**0.78**	**0.862**	**0.732**	**0.992**
Frequency in Kenya	0.102	0.102	0.122	0.061	0.184	0	0.163
Kenya	T	S	G	V	H	I	R
Frequency in Pakistan	0.049	0.008	0.195	0.211	0.122	0.244	0.008
Frequency in Kenya	**0.673**	**0.898**	**0.878**	**0.898**	**0.653**	**0.959**	**0.837**
HXB2 Position	303	332	357	370	372	375	383

**Table 3:** Unique HLA Anchoring Residues and restrictions. The HLA anchoring residue analysis of the possible unique epitopes, their restricted HLA, and their anchoring residues in HIV-1 sub-subtype A1 in Pakistan and Kenyan reference datasets. Columns 1–4 and columns 5–8 represent the data from HIV-1 sub-subtype A1 from Pakistan and the Kenyan reference dataset, respectively. The amino acid in bold and underline represents the signature amino acids between the strains.

										
**Pakistan**	**Restricted HLA**	**HXB2 Position**	**Epitope**	**Anchors**	**Kenya**	**Restricted HLA**	**HXB2 Position**	**Epitope**	**Anchors**	
	B*5103, Cw*0601, Cw*0602	295-303	DYVDRFFK**V**	........V		DRB1*0301 or DRB3*0201	300-303	FFK**T**	F..T	
	DPA1*0102, DPB1*0201	296-303	YVDRFFK**V**	Y...F..V		B*7801	325-333	NANPDCK**S**I	.A.....S.	
	DRB1*1501	297-303	VDRFFK**V**	V..F..V		B*7801	356-364	P**G**HKARVLA	.G.......	
	DPA1*0201/DPB1*0401	297-306	VDRFFK**V**LRA	V.....V..A		A*0201, A*0202, A*0214	362-370	VLAEAMSQ**V**	.L......V	
	DRB1*0901 or DRB4*0101	300-303	FFK**V**	F..V		B*5103, Cw*0601, Cw*0602			........V	
	DRB1*0401 or DRB4*0101	300-308	FFK**V**LRAEQ	F..V.RA.Q		DRB1*1501 or DRB5*0101	363-372	LAEAMSQ**V**Q**H**	L........H	
	A*0206, B*3501	302-310	K**V**LRAEQAT	.V.......		B*1517	367-375	MSQ**V**Q**H**TN**I**	.S......I	
	DRB1*0401	303-311	**V**LRAEQATQ	V.......Q		B*3801, B*5101, B*5103, Cw*0601, Cw*0602			........I	
	DRB1*0401 or DRB4*0101			V..A.QA.Q		A*0206	369-377	Q**V**Q**H**TN**I**MM	.V.......	
	DQA1*0102, DQB1*0602	352-360	GVGGP**S**HKA	.....S..A		A*3001			.V......M	
	DQA1*0301, DQB1*0302	357-365	**S**HKARVLAE	S.......E		DRB1*0301 or DRB3*0201	370-373	**V**Q**H**T	V..T	
	B*7801	363-371	LAEAMSQ**A**Q	.A.....A.		DRB1*0401	370-378	**V**Q**H**TN**I**MMQ	V.......Q	
	DQA1*0301, DQB1*0301	365-370	EAMSQ**A**	E..S.A		DRB1*1501 or DRB5*0101	370-379	**V**Q**H**TN**I**MMQR	V........R	
	A*3004, B*1502	367-375	MSQ**A**Q**Q**TN**M**	........M		DQA1*0301/DQB1*0301	371-375	Q**H**TN**I**	..T.I	
	B*5101, Cw*0303, Cw*0305-0306, Cw*0308-0309, Cw*0801-Cw*0806, Cw*1502, Cw*1503, Cw*1506, Cw*1507	369-377	Q**A**Q**Q**TN**M**MM	.A......M		A*2601, A*2602, A*2603	374-382	N**I**MMQRGNF	.I......F	
	B*7801			.A.......		DRB1*0301 or DRB3*0201	375-378	**I**MMQ	I..Q	
	A*0206	371-379	Q**Q**TN**M**MMQR	.Q.......		DRB1*0801	375-379	**I**MMQR	I...R	
	B*1512	374-382	N**M**MMQRGNF	.M......F		A*3101, A*3303	375-383	**I**MMQRGNF**R**	........R	
	B*2703			.M.......		B*2703, DPB1*0301	382-390	F**R**GQKRIKC	.R.......	
	DRB5*0101	375-383	**M**MMQRGNF**K**	M..Q....K		Signature Amino Amino Acid: Bold and underline				
									

## Data Availability

The data is available within the manuscript or its supplementary files.

## Supplementary Materials

The following supporting information can be downloaded at: https://www.mdpi.com/article/10.3390/v14102307/s1, Table S1: List of accession number of HIV-1 Gag sequences from Pakistan used in this study.; Table S2: Profile of the people living with HIV-1 (PLHV-1) in Pakistan recruited for this study.; Table S3: The list of accession numbers of all Kenyan HIV-1 subtype A1 sequences from the database used to construct the Kenyan HIV-1 subtype A1 tree.; Table S4: The list of net charges and total charges.; Table S5: List of anchoring residues in HIV-1 subtype A1 gag protein from Kenya and Pakistan.; Table S6: The list of accession numbers along with biophysical properties difference of the sequences different between Kenyan and Pakistani HIV-1 subtype A1.; Figure S1. Genotyping of HIV-1 subtype A1.; Figure S2. Clustering pattern of Kenyan reference dataset sequence with all available Kenyan sequences from the Los Alamos HIV Database.; Figure S3. Amino acid variations within Pakistani and Kenyan cohorts.; Figure S4. Sites under selection pressure in Pakistani and Kenyan reference subtype A1.
