# Supplementary material for: Phylogenetic Characterization of HIV-1 Sub-Subtype A1 in Karachi, Pakistan
Source: Viruses. 2022 Oct 20;14(10):2307. doi: 10.3390/v14102307 (PMC9611109; doi:10.3390/v14102307)

SUPPLEMENTARY DATA

Table S1.

List of accession number of HIV-1 Gag sequences from Pakistan used in this study.

Table S2.

Profile of the people living with HIV-1 (PLHV-1) in Pakistan recruited for this study. The participants belong to the following high-risk communities: people who inject drugs (PWID) (N=43), men who have sex with men (MSM) (N=10), heterosexual (HET) (N=3), and spouses of Infected men (SP) (N=05). Few participants with no information for high-risk behavior as marked as not available (NA).

Table S3.

The list of accession numbers of all Kenyan HIV-1 subtype A1 sequences from the database was used to construct the Kenyan HIV-1 subtype A1 tree.

Table S4.

The list of net charges and total charges. A) The list of accessions of Pakistani Subtype A1 gag protein sequences with their respective net charge (NC) and total charge (TC). B) The list of accession numbers of Kenyan Subtype A1 strain sequences with their respective net charge (NC) and total charge (TC).

Table S5.

List of anchoring residues in HIV-1 subtype A1 gag protein from Kenya and Pakistan. Columns 1-4 represent the predicted epitope, the position in reference to the HXB2, restricting HLA types, and restricted HLA amino acid anchors in HIV-1 subtype A. A1) The list of anchoring residues in HIV-1 subtype A1 gag protein at position 303 HXB2 in Pakistan. A2) The list of anchoring residues in HIV-1 subtype A1 gag protein at position 303 HXB2 in Kenya. B1) The list of anchoring residues in HIV-1 subtype A1 gag protein at position 332 HXB2 in Pakistan. B2) The list of anchoring residues in HIV-1 subtype A1 gag protein at position 332 HXB2 in Kenya. C1) The list of anchoring residues in HIV-1 subtype A1 gag protein at position 357 HXB2 in Pakistan. C2) The list of anchoring residues in HIV-1 subtype A1 gag protein at position 357 HXB2 in Kenya. D1) The list of anchoring residues in HIV-1 subtype A1 gag protein at positions 370, 372 and 375 HXB2 in Pakistan. D2) The list of anchoring residues in HIV-1 subtype A1 gag protein at positions 370, 372 and 375 HXB2 in Kenya. E1) The list of anchoring residues in HIV-1 subtype A1 gag protein at position 383 HXB2 in Pakistan. E2) The list of anchoring residues in HIV-1 subtype A1 gag protein at position 383 HXB2 in Kenya. The unique epitopes identified in Kenyan and Pakistani HIV-1 subtype A1 sequences are highlighted in grey. The unique restricting HLA types and HLA anchoring amino acids between Kenyan and Pakistani HIV-1 subtype A1 are represented in blue font.

Table S6. The list of accession numbers along with biophysical properties difference of the sequences different between Kenyan and Pakistani HIV-1 subtype A1. The colors blue and grey represents unique positions, 312, 319, 331, 372, 273, 383, and 402, in Pakistani and Kenyan sequences. The amino acid difference in each position for Pakistani and Kenyan sequences is given

in column 3, whereas the number of amino acids for each position is given in column 4. The amino acids are represented as arginine (R), lysine (K), glutamic acid (E), aspartic acid (D), and histidine (H). The potential N-linked glycosylation sites (PNGs) were only found in Kenyan HIV-1 subtype A1 sequences.

Figure S1. Genotyping of HIV-1 subtype A1. HIV-1 subtype A1 sequences from Pakistan and subtype A1 reference sequences from Los Alamos Database (https://www.hiv.lanl.gov/) are represented with blue and purple branches, respectively.

Figure S2. Clustering pattern of Kenyan reference dataset sequence with all available Kenyan sequences from the Los Alamos HIV Database. The Blue highlighted area in the maximum likelihood tree represents the clusters of database sequences containing Kenyan reference dataset sequences.

Figure S3. Amino acid variations within Pakistani and Kenyan cohorts. Acidic, Basic, Polar, and Hydrophobic amino acids are represented in Red, Blue, Green, and purple colors. The dominant amino acids are shown as web logos for A) Kenyan Reference Data set and B) the Pakistani dataset.

Figure S4. Sites under selection pressure in Pakistani and Kenyan reference subtype A1. A) Mean dN/dS ration of HV-1 subtype A1 from Pakistan. B) Mean dN/dS ration of HIV-1 subtype A1 from Kenyan reference dataset. X-axis and Y-axis represented the HXB2 position of HIV-1 Gag protein and mean dN/dS ratio, respectively. The red dotted line defines the threshold for positive selection pressure.

Table S1. List of accession number of HIV-1 Gag sequences from Pakistan used in this study.

Accession numbers for the Pakistani HIV-1 gag sequences used in the study	
**Sequences from the PLHIV part of our study cohort**	KY435514-KY435538, KY435540-KY435548, KY435564-KY435566, MH819260, MH819262-MH819276, MH819261 and MH819277-MH819283
**Sequences deposited previously to the Los Alamos HIV Database from Pakistan**	GU376767, GU376770, GU376772-GU376774, GU376776-GU376779, GU376781, GU376783-GU376785, GU376788-GU376792, JF804693-JF804721, JF804723-JF804726, JF804728-JF804737, JF804739-JF804741, JF804743-JF804744, KX232594-KX232595, KX232597, KX232600, KX232606, KX232607, KX232609-KX232611, KX232614, KX232618-KX232621, KX232624 and KX232626**.**

Table S2. Patient profile of the recruited people living with HIV-1 (PLHV-1) in Pakistan.

High-Risk Behaviors	PWID	MSM	HET	SP	NA
No. of Patients	N=42	N=10	N=03	N=05	N=02

CD4 cell count	Low CD4 cells count (<350 cells/mm3)	High CD4 cells count (>350 cells/mm3)
No. of Patients	N=10	N=51

Viral Load (IU/ml)	Detectable Viral Load (> 400 IU/ml)	Undetectable Viral Load
No. of Patients	N=30	N=13

Table S3. The list of accession numbers of all Kenyan HIV-1 subtype A1 sequences from the database used to construct the Kenyan HIV-1 subtype A1 phylogenetic tree.

Accession numbers of all available Kenyan subtype A1 sequences in the Los Alamos Sequence Database
AF004885, AF184374, AF184418-AF184424, AF184426-AF184432, AF184434, AF184436-AF184439, AF184441, AF184443-AF184452, AF184454-AF184457, AF184459-AF184461, AF184463, AF457052-AF457053, AF457055, AF457063, AF457065-AF457070, AF457079-AF457081, AF457083-AF457084, AF457086, AY322184-AY322185, AY322190, AY322193, AY492753, AY492761, AY492765, AY492777, AY492780, AY772953-AY772955, AY772958, AY772968-AY772971, AY772985, DQ367262-DQ367266, DQ367268-DQ367269, DQ367271-DQ367275, DQ367277-DQ367278, DQ367280-DQ367284, DQ367286-DQ367288, DQ36729-DQ367296, EU110085, EU110087-EU110088, EU110092, EU110094-EU110095, EU110097, FJ623475-FJ623486, FJ623488, GQ429817-GQ429866, GQ429868-GQ429891, GQ429893-GQ429941, GQ430033-GQ430034, GQ430036-GQ430090, GQ430176-GQ430206, GQ430208, GQ430210, GQ430213-GQ430219, GQ430222-GQ430223, GQ430225, GQ430227, GQ430230, GQ430293, GQ430295- GQ430318, GQ430320- GQ430324, GQ430355-GQ430356, GQ430358-GQ430445, GQ430448-GQ430478, GQ430480-GQ430487, GQ430489-GQ430508, GQ430510-GQ430644, GQ430674-GQ430701, GQ430703-GQ430706, GQ430708-GQ430737, GQ430768-GQ430824, GQ430826-GQ430892, GQ430929, GQ430931, GQ430933-GQ430934, GQ430941, GQ430949, GQ430951, GQ430955, GQ430988-GQ430991, GQ430993-GQ430995, GQ430997-GQ431022, GQ431024-GQ431033, GQ431220-GQ431229, GQ431232-GQ431234, GQ431236-GQ431237, GQ431241-GQ431242, GQ431244-GQ431245, GQ431248, GQ431310-GQ431325, GQ431357-GQ431359, GQ431361-GQ431368, GQ431370-GQ431375, GQ431377-GQ431386, GQ431388, GQ431391-GQ431392, GQ431396, GQ431398, GQ431401, GQ431404, GQ431406-GQ431407, GQ431415-GQ431447, GQ431661-GQ431692, GQ431724-GQ431749, GQ431783, GQ431790, GQ431844-GQ431852, GQ431854-GQ431867,

GQ431869-GQ431875, GQ431908-GQ431949, GQ431951-GQ431964, GQ431966, GQ431968, GQ431970, GQ431972-GQ431973, GQ431975, GQ431977-GQ431978, GQ431981, GQ431983-GQ431984, GQ431986-GQ432064, GQ432094, GQ432097-GQ432099, GQ432101-GQ432103, GQ432105-GQ432106, GQ432109-GQ432112, GQ432114, GQ432116-GQ432120, GQ432122, GQ432124, GQ432127, GQ432138, GQ432140-GQ432141, GQ432143, GQ432150-GQ432182, GQ432184-GQ432208, GQ432238, GQ432240, GQ432244, GQ432246-GQ432247, GQ432250-GQ432252, GQ432255-GQ432257, GQ432260, GQ432262, GQ432264, GQ432294-GQ432306, GQ432308-GQ432336, GQ432338-GQ432389, GQ432391-GQ432412, GQ432414-GQ432415, GQ432417-GQ432419, GQ432422-GQ432424, GQ432426, GQ432428-GQ432436, GQ432438-GQ432441, GQ432443, GQ432445-GQ432479, GQ432481-GQ432491, GQ432493-GQ432529, GQ432562-GQ432593, GQ432595-GQ432625, GQ432627-GQ432633, GQ432636-GQ432638, GQ432640-GQ432646, GQ432648-GQ432653, GQ432655-GQ432664, GQ432666-GQ432668, GQ432670-GQ432693, GQ432696-GQ432698, GQ432701-GQ432747, GQ432749-GQ432758, GQ432760-GQ432764, GQ432766, GQ432768, GQ432772, GU245696, GU245698-GU245702, GU245705-GU245710, GU245712-GU245715, GU245721, GU245725, GU245727, GU245730, GU245732, GU245734, GU245736-GU245737, GU245741-GU245743, GU245745-GU245746, GU245748-GU245750, GU245758, GU245760-GU245763, KF716474-KF716475, KR020057, KR020059, KR020061, KR020064-KR020069, KR020073, KR020075-KR020076, KR020078-KR020082, KR020085-KR020086, KR020090, KR020092, KR020096-KR020098, KR020100-KR020102, KR781619-KR781620, KR781622-KR781625, KR781627-KR781628, KR781630-KR781631, KR781641, KR781646, KT022360-KT022361, KT022363-KT022365, KT022368-KT022370, KT022372-KT022378, KT022380-KT022384, KU921861-KU921867, KU921885-KU921888, KU921893-KU921903, KU921911-KU921917, KU921926-KU921938, KU921948-KU921961, KU921975-KU921978, KU921983-KU921986, KU921991-KU921994, KU922008-KU922010, KU922017-KU922019, KU922046, KU922051-KU922055, KU922058, KU922063-KU922064, KU922069-KU922076, KU922087-KU922089, KX233975-KX233977, KX233980,

KX233989, KX233991, KX233993, KX233996-KX233998, KX234002-KX234003, KX234010-KX234011, KX234013, KX234016-KX234017, KX234021, KX234025, KX234028-KX234030, KX234033, KX234035-KX234040, KX234042, KX234044-KX234045, KX234048-KX234049, KX234052, KX234054-KX234055, KX234061, KX234066-KX234068, KX234070-KX234073, KX234076-KX234077, KX302403-KX302404, KX302408, KX302414-KX302415, KX302427-KX302428, KX302431-KX302434, KX302448-KX302449, KX302472-KX302474, KX302478, KX302489, KX302490-KX302491, KX302497-KX302498, KX302507, KX302512, KX302514-KX302515, KX302526, KX302529-KX302532, KX302545-KX302547, KX302551-KX302554, KX302569-KX302571, KX302580-KX302581, KX302584, KX302592-KX302593, KY351787-KY351798, L11773-L11775.

Table S4. The list of Net charges and total charge

A											
**S#**	**Sequence**	**NC**	**TC**	**S#**	**Sequence**	**NC**	**TC**	**S#**	**Sequence**	**NC**	**TC**
1	KY435514	13	37	42	MH819283	13	37	83	JF804710	13	37
2	KY435516	13	37	43	KY435533	13	37	84	JF804707	13	37
3	MH819262	13	37	44	KY435518	13	37	85	JF804701	13	37
4	KY435564	13	37	45	KY435528	13	37	86	JF804732	13	37
5	MH819279	11	35	46	KY435543	13	37	87	JF804718	13	37
6	KY435524	13	37	47	JF804729	13	37	88	JF804735	12	36
7	KY435517	13	37	48	JF804693	12	36	89	KX232594	13	37
8	KY435519	13	37	49	JF804696	13	37	90	KX232595	13	37
9	MH819263	13	37	50	JF804724	13	37	91	KX232597	13	37
10	KY435520	13	37	51	JF804728	13	37	92	KX232600	13	37
11	KY435523	14	38	52	JF804734	13	37	93	KX232606	13	37
12	KY435535	13	37	53	JF804733	13	37	94	KX232607	12	38
13	KY435566	15	37	54	JF804697	13	37	95	KX232609	13	37
14	KY435515	13	37	55	JF804694	13	37	96	KX232610	13	37
15	KY435521	13	37	56	JF804723	13	37	97	KX232611	13	37
16	MH819265	13	37	57	JF804695	13	37	98	KX232614	13	37
17	KY435530	13	37	58	JF804726	13	37	99	KX232618	13	37
18	MH819281	12	38	59	JF804725	13	37	100	KX232619	13	37
19	MH819266	16	38	60	JF804708	13	37	101	KX232620	13	37
20	KY435536	13	37	61	JF804709	13	37	102	KX232621	13	37
21	KY435542	13	37	62	JF804702	13	37	103	KX232624	13	37
22	KY435522	13	37	63	JF804730	13	37	104	KX232626	13	37
23	KY435525	13	37	64	JF804720	13	37	105	GU376767	13	37
24	KY435527	13	37	65	JF804700	13	37	106	GU376770	13	37
25	MH819267	13	37	66	JF804704	13	37	107	GU376772	13	37
26	KY435534	13	37	67	JF804711	13	37	108	GU376773	13	37
27	MH819269	13	37	68	JF804715	13	37	109	GU376774	13	37
28	MH819272	13	37	69	JF804737	12	36	110	GU376776	13	37
29	KY435541	13	37	70	JF804719	13	37	111	GU376777	13	37
30	KY435537	13	37	71	JF804697	13	37	112	GU376778	13	37
31	MH819273	13	37	72	JF804699	13	37	113	GU376779	13	37
32	MH819282	12	36	73	JF804706	12	36	114	GU376781	13	37
33	KY435526	13	37	74	JF804713	12	36	115	GU376783	13	37
34	KY435538	13	37	75	JF804714	13	37	116	GU376784	13	37
35	MH819274	13	37	76	JF804716	13	37	117	GU376785	13	37
36	KY435529	13	37	77	JF804717	13	37	118	GU376788	13	37
37	MH819280	13	37	78	JF804736	12	36	119	GU376789	13	37
38	MH819275	13	37	79	JF804703	13	37	120	GU376790	13	37
39	KY435531	13	37	80	JF804731	13	37	121	GU376791	13	37
40	MH819276	13	37	81	JF804721	13	37	122	GU376792	13	37
41	KY435532	13	37	82	JF804712	12	36	123	JF804705	13	37

B

S#	Sequence	NC	TC	S#	Sequence	NC	TC
1	AF184453	13	37	27	GQ429867	13	37
2	AF184458	13	37	28	KR781626	13	37
3	AF184435	13	37	29	AY492759	13	37
4	AF184442	13	37	30	GQ432183	13	37
5	AF184462	13	37	31	GQ432420	13	37
6	AF184440	13	37	32	GQ432635	13	37
7	L11768	13	37	33	GQ432759	13	37
8	KR781621	13	37	34	AY772986	13	37
9	KX234022	13	37	35	GQ432480	13	37
10	AF539405	13	37	36	DQ367289	13	37
11	GQ432307	13	37	37	DQ367290	13	37
12	GQ432337	13	37	38	KX302407	13	37
13	GQ430209	12	36	39	AF457089	13	37
14	GQ430294	13	37	40	GQ430357	13	37
15	GQ431965	13	37	41	GQ431023	13	37
16	GQ432033	13	37	42	GQ430035	13	37
17	GQ432390	13	37	43	KT022367	13	37
18	GQ430393	12	36	44	FJ623487	13	37
19	GQ430447	13	37	45	KR020077	12	36
20	GQ430479	13	37	46	KR020056	13	37
21	GQ430564	13	37	47	KR020105	13	37
22	GQ430825	13	37	48	KR020074	13	37
23	AY492766	13	37	49	KR020087	13	37
24	GQ431950	13	37	50	KX302503	13	37
25	GQ432249	13	37	51	FJ623491	13	37
26	GQ432594	13	37				

Table S5. List of anchoring residues in HIV-1 subtype A1 gag protein from Kenya and Pakistan.

A1	Gag 303 HXB2 Position		
Pakistani HIV-1 Subtype A1 Strain			
**Epitope**	**HXB2 Position**	**Restricted HLA**	**Anchors**
DYVDRFFKV	2358-303	B*5103, Cw*0601-0602	V
		Cw*0401	.Y.......
YVDRFFKV	2359-303	DPA1*0102/DPB1*0201	Y...F..V
YVDRFFKVL	2359-304	A*0205, A*0206, A*0214, A*2602-2603, A*3001, A*6802, A*6901, B*0702, B*3501, B*3503	.V......L
		A*0205, A*3004, B*3801, Cw*0301, Cw*0601-0602, Cw*0702	L
		A*0206, B*3501	.V.......
VDRFFKV	2360-303	DRB1*1501	V..F..V
VDRFFKVLRA	2360-306	DPA1*0201/DPB1*0401	V.....V..A
VDRFFKVLR	2360-305	A*1101, A*3101, A*3303	........R
		B*0801	..R......
DRFFKVLRA	2361-306	B*2703, B*2705, DPB1*0301	.R.......
		DQA1*0102/DQB1*0602	.....V..A
		DQB1*0602	D.FF.V..A
FFKV	300-303	DRB1*0901 or DRB4*0101	F..V
FFKVLRAEQ	300-308	B*0801	..K......
		DRB1*0401	F.......Q
		DRB1*0401 or DRB4*0101	F..V.RA.Q
FFKVLRAEQA	300-309	DPA1*0201/DPB1*0401	F.....A..A
FKVLR	301-305	DRB1*0801	F...R
FKVLRAEQA	301-309	B*2705	.K.......
		DQA1*0102/DQB1*0602	.....A..A
		DQB1*0602	F.VL.A..A
		DRB1*0101	F..L.A..A
KVLRAEQAT	302-310	A*0206, B*3501	.V.......
VLRAEQATQ	303-311	B*0801	..R......
		DRB1*0401	V.......Q
		DRB1*0401 or DRB4*0101	V..A.QA.Q

A2	Gag 303 HXB2 Position		
Kenyan HIV-1 Subtype A1 Strain			
Epitope	HXB2 Position	Restricted HLA	Anchors
DYVDRFFKT	2358-303	Cw*0401	.Y.......
YVDRFFKTL	2359-304	A*0205, A*0206, A*0214, A*2602-2603, A*3001, A*6802, A*6901, B*0702, B*3501, B*3503	.V......L
		A*0205, A*3004, B*3801, Cw*0301, Cw*0601-0602, Cw*0702	L
		A*0206, B*3501	.V.......
VDRFFKTLR	2360-305	A*1101, A*3101, A*3303	........R
		B*0801	..R......
DRFFKTLRA	2361-306	B*2703, B*2705, DPB1*0301	.R.......
		DQA1*0102/DQB1*0602	.....T..A
		DQB1*0602	D.FF.T..A
FFKT	300-303	DRB1*0301 or DRB3*0201	F..T
FFKTLRAEQ	300-308	B*0801	..K......
		DRB1*0401	F.......Q
FFKTLRAEQA	300-309	DPA1*0201/DPB1*0401	F.....A..A
FKTLR	301-305	DRB1*0801	F...R
FKTLRAEQA	301-309	B*2705	.K.......
		DQA1*0102/DQB1*0602	.....A..A
		DQB1*0602	F.TL.A..A
		DRB1*0101	F..L.A..A
TLRAEQATQ	303-311	B*0801	..R......

B1	Gag 332 HXB2 Position		
Pakistani HIV-1 Subtype A1 Strain			
**Epitope**	**HXB2 Position**	**Restricted HLA**	**Anchors**
NANPDCKTI	325-333	B*3501, B*7801	.A.......
		B*3501, B*5101-5103	.A......I
		B*3801, B*5101, B*5103, Cw*0301, Cw*0601-0602	I
ANPDCKTIL	326-334	A*0205, A*3004, B*3801, Cw*0301, Cw*0601-0602, Cw*0702	L
		B*0703	.N......L
		Cw*0102	..P.....L
NPDCKTILR	327-335	A*1101, A*3101, A*3303	........R
		A*6601	.P......R
		B*0703, B*0705, B*3501, B*5301, B*5401, B*5501-5502, B*5601, B*6701, B*7801, Cw*0401	.P.......
PDCKTILRA	328-336	DQA1*0102/DQB1*0602	.....I..A
DCKTIL	329-334	DQA1*0301/DQB1*0301	D..T.L
DCKTILRAL	329-337	A*0205, A*3004, B*3801, Cw*0301, Cw*0601-0602, Cw*0702	L
		B*0801	..K......
		DQA1*0102/DQB1*0602	.....L..L
CKTIL	330-334	DQA1*0301/DQB1*0301	..T.L
CKTILRALG	330-338	B*2705	.K.......

B2	Gag 332 HXB2 Position		
Kenyan HIV-1 Subtype A1 Strain			
Epitope	HXB2 Position	Restricted HLA	Anchors
NANPDCKSI	325-333	B*3501, B*7801	.A.......
		B*3501, B*5101-5103	.A......I
		B*3801, B*5101, B*5103, Cw*0301, Cw*0601-0602	I
		B*7801	.A.....S.
ANPDCKSIL	326-334	A*0205, A*3004, B*3801, Cw*0301, Cw*0601-0602, Cw*0702	L
		B*0703	.N......L
		Cw*0102	..P.....L
NPDCKSILR	327-335	A*1101, A*3101, A*3303	........R
		A*6601	.P......R
		B*0703, B*0705, B*3501, B*5301, B*5401, B*5501-5502, B*5601, B*6701, B*7801, Cw*0401	.P.......
PDCKSILRA	328-336	DQA1*0102/DQB1*0602	.....I..A
DCKSIL	329-334	DQA1*0301/DQB1*0301	D..S.L
DCKSILRAL	329-337	A*0205, A*3004, B*3801, Cw*0301, Cw*0601-0602, Cw*0702	L
		B*0801	..K......
		DQA1*0102/DQB1*0602	.....L..L
CKSIL	330-334	DQA1*0301/DQB1*0301	..S.L
CKSILRALG	330-338	B*2705	.K.......

C1	Gag 357 HXB2 Position		
Pakistani HIV-1 Subtype A1 Strain			
Epitope	HXB2 Position	Restricted HLA	Anchors
CQGVGGPSH	350-358	A*0206	.Q.......
QGVGGPSHK	351-359	A*1101	........K
		B*7801	.G.......
GVGGPSHKA	352-360	A*0206, B*3501	.V.......
		DQA1*0102/DQB1*0602	.....S..A
VGGPSHKAR	353-361	A*1101, A*3101, A*3303	........R
		B*7801	.G.......
			.G.....A.
		DRB1*0401 or DRB4*0101	V..P.HK.R
GGPSHKARV	354-362	B*5101-5103	.G......V
		B*5103, Cw*0601-0602	V
		B*7801	.G.......
GPSHKARVL	355-363	A*0205, A*3004, B*3801, Cw*0301, Cw*0601-0602, Cw*0702	L
		B*0702-0703, B*0705, B*3501, B*3503, B*3909, B*4201, B*5101, B*5301, B*5601, B*8101, Cw*0401-0406, Cw*0603, Cw*0703	.P......L
		B*0703, B*0705, B*3501, B*5401, B*5501-5502, B*5601, B*6701, B*7801, Cw*0401	.P.......
		DQA1*0102/DQB1*0602	.....A..L
SHKARVLAE	357-365	B*0801	..K.R....
		B*0801	..K......
		DQA1*0301/DQB1*0302	S.......E

C2	Gag 357 HXB2 position		
Kenyan HIV-1 Subtype A1 Strain			
Epitope	HXB2 Position	Restricted HLA	Anchors
CQGVGGPGH	350-358	A*0206	.Q.......
QGVGGPGHK	351-359	A*1101	........K
		B*7801	.G.......
GVGGPGHKA	352-360	A*0206, B*3501	.V.......
VGGPGHKAR	353361	A*1101, A*3101, A*3303	........R
		B*7801	.G.......
			.G.....A.
		DRB1*0401 or DRB4*0101	V..P.HK.R
GGPGHKARV	354-362	B*5101-5103	.G......V
		B*5103, Cw*0601-0602	V
		B*7801	.G.......
GPGHKARVL	355-363	A*0205, A*3004, B*3801, Cw*0301, Cw*0601-0602, Cw*0702	L
		B*0702-0703, B*0705, B*3501, B*3503, B*3909, B*4201, B*5101, B*5301, B*5601, B*8101, Cw*0401-0406, Cw*0603, Cw*0703	.P......L
		B*0703, B*0705, B*3501, B*5301, B*5501-5502, B*5601, B*6701, B*7801, Cw*0401	.P.......
		DQA1*0102/DQB1*0602	.....A..L
PGHKARVLA	356-364	B*7801	.G.......
GHKARVLAE	357-365	B*0801	..K.R....
			..K......

D1	Gag 370, 372, 375 HXB2 Position		
Pakistani HIV-1 Subtype A1 Strain			
Epitope	HXB2 Position	Restricted HLA	Anchors
LAEAMSQA	363-370	DPA1*0102/DPB1*0201	L...M..A
LAEAMSQAQ	363-371	A*6901	.A......Q
		B*3501, B*7801	.A.......
		B*7801	.A.....A.
		DRB1*0401	L.......Q
		DRB1*0401 or DRB4*0101	L..A.SQ.Q
AEAMSQAQQ	364-372	B*18	.E.......
		B*3701	.E.....QQ
EAMSQA	365-370	DQA1*0301/DQB1*0301	E..S.A
EAMSQAQQT	365-373	B*3501, B*7801	.A.......
		DQA1*0102/DQB1*0602	.....A..T
		DQB1*0602	E.MS.A..T
AMSQA	366-370	DQA1*0301/DQB1*0301	..S.A
AMSQAQQTN	366-374	B*2703	.M.......
MSQAQQTNM	367-375	A*3004, B*1502, Cw*0301	........M
		B*1516	.S......M
		DRB1*0401 or DRB4*0101	M..A.QT.M
SQAQQTNMM	368-376	A*0206	.Q.......
		A*0214, B*1502, B*3902, Cw*0704	.Q......M
		A*3004, B*1502, Cw*0301	........M
		B*5201	.......MM
QAQQTNMMM	369-377	A*0214, A*6901, B*1502, B*3501, B*3503, B*5101, Cw*0303-0306, Cw*0308-Cw*0309, Cw*0801-Cw*0806,	.A......M
		A*3004, B*1502, Cw*0301	........M
		B*3501, B*7801	.A.......
		B*5201	.......MM
AQQTNMMMQ	370-378	A*0206	.Q.......
QQTNMMMQR	371-379	A*0206	.Q.......
		A*1101, A*3101, A*3303	........R
NMMMQRGNF	374-382	A*0301, A*3001, B*1512, B*3503, B*4402-4403, B*30901	.M......F
		B*1502, B*3801, B*30901, B*5101, B*5103, Cw*0301, Cw*0702	........F
		B*2703	.M.......

D2		Gag 370, 372, 375 HXB2 Position	
Kenyan HIV-1 Subtype A1 Strain			
Epitope	HXB2 Position	Restricted HLA	Anchors
VLAEAMSQV	362-370	A*0201-0202, A*0214	.L......V
		B*5103, Cw*0601-0602	V
LAEAMSQV	363-370	DPA1*0102/DPB1*0201	L...M..V
LAEAMSQVQ	363-371	A*6901	.A......Q
		B*3501, B*7801	.A.......
		DRB1*0401	L.......Q
		DRB1*0401 or DRB4*0101	L..A.SQ.Q
LAEAMSQVQH	363-372	DRB1*1501 or DRB5*0101	L........H
AEAMSQVQH	364-372	B*18	.E.......
		B*3701	.E.....QH
EAMSQVQHT	365-373	B*3501, B*7801	.A.......
		DQA1*0102/DQB1*0602	.....V..T
		DQB1*0602	E.MS.V..T
AMSQV	366-370	DQA1*0301/DQB1*0301	..S.V
AMSQVQHTN	366-374	B*2703	.M.......
MSQVQHTNI	367-375	B*1516-1517	.S......I
		B*3801, B*5101, B*5103, Cw*0301, Cw*0601-0602	I
		DRB1*0401 or DRB4*0101	M..V.HT.I
SQVQHTNIM	368-376	A*0206	.Q.......
		A*0214, B*1502, B*3902, Cw*0704	.Q......M
		A*3004, B*1502, Cw*0301	........M
		B*5201	.......IM
QVQHTNIMM	369-377	A*0206, B*3501	.V.......
		A*0214, A*2602, A*2603,A*3001, A*6901, B*1502, B*3501, B*3503	.V......M
		A*3004, B*1502, Cw*0301	........M
		B*5201	.......MM
VQHT	370-373	DRB1*0301 or DRB3*0201	V..T
VQHTNIMMQ	370-378	A*0206	.Q.......
		DRB1*0401	V.......Q
VQHTNIMMQR	370-379	DRB1*1501 or DRB5*0101	V........R
QHTNI	371-375	DQA1*0301/DQB1*0301	..T.I
QHTNIMMQR	371-379	A*1101, A*3101, A*3303	........R
NIMMQRGNF	374-382	A*0301, A*2601-A*2603, A*3001, B*3503, B*4402-4403, B*30901	.I......F
		B*1502, B*3801, B*30901, B*5101, B*5103, Cw*0301, Cw*0702	........F
IMMQ	375-378	DRB1*0301 or DRB3*0201	I..Q
IMMQR	375-379	DRB1*0801	I...R

E1	Gag 383 HXB2 Position		
Pakistani HIV-1 Subtype A1 Strain			
**Epitope**	**HXB2 Position**	**Restricted HLA**	**Anchors**
**M**MMQRGNF**K**	375-383	A*0301	-M------K
		A*1101	--------K
		B*2703	-M-------
		DRB5*0101	M--Q----K
MMQRGNF**K**G	376-384	B*2703	-M-------
MQRGNF**K**GQ	377-385	A*0206	-Q-------
		B*0801	--R------
QRGNF**K**GQK	378-386	A*1101	--------K
		B*2703, B*2705, DPB1*0301	-R-------
		B*2705	-R------K
RGNF**K**GQKR	379-387	A*1101, A*3101, A*3303	--------R
		B*7801	-G-------
			-G-----K-
GNF**K**GQKRI	380-388	B*3801, B*5101, B*5103, Cw*0301, Cw*0601-0602	--------I
NF**K**GQKRIK	381-389	A*1101	--------K
		B*0801	--K------
F**K**GQ	382-385	DRB1*0301 or DRB3*0201	F--Q
F**K**GQK	382-386	DRB1*0801	F---K
F**K**GQKRIK	382-389	DRB1*1101 or DRB3*0202	F----R-K
F**K**GQKRIKC	382-390	B*2705	-K-------
**K**GQKRIKCF	383-391	B*1502, B*3801, B*30901, B*5101, B*5103, Cw*0301, Cw*0702	--------F
		B*5103, B*5801	-G------F
		B*7801	-G-------

E2	Gag 383 HXB2 Position		
Kenyan HIV-1 Subtype A1 Strain			
**Epitope**	**HXB2 Position**	**Restricted HLA**	**Anchors**
**I**MMQRGNF**R**	375-383	A*0301	.M......R
		A*1101, A*3101, A*3303	........R
		B*2703	.M.......
MMQRGNF**R**G	376-384	B*2703	.M.......
MQRGNF**R**GQ	377-385	A*0206	.Q.......
		B*0801	..R......
QRGNF**R**GQK	378-386	A*1101	........K
		B*2703, B*2705, DPB1*0301	.R.......
		B*2705	.R......K
RGNF**R**GQKR	379-387	A*1101, A*3101, A*3303	........R
		B*7801	.G.......
			.G....K..
GNF**R**GQKRI	380-388	B*3801, B*5101, B*5103, Cw*0301, Cw*0601-0602	I
NF**R**GQKRIK	381-389	A*1101	........K
		B*0801	..R......
F**R**GQ	382-385	DRB1*0301 or DRB3*0201	F..Q
F**R**GQK	382-386	DRB1*0801	F...K
F**R**GQKRIK	382-389	DRB1*1101 or DRB3*0202	F....R.K
F**R**GQKRIKC	382-390	B*2703, B*2705, DPB1*0301	.R.......
**R**GQKRIKCF	383-391	B*1502, B*3801, B*30901, B*5101, B*5103, Cw*0301, Cw*0702	........F
		B*5103, B*5801	.G......F
		B*7801	.G.......

Table S6. The list of accession numbers of the sequences of highlighted position in biophysical properties analysis.

Position	Acession	Amino Acid	Count
312	KX232594, KY435520, KY435537	D	3
	AF184458, AY492759, FJ623487, FJ623491, GQ431950, GQ432635, KR781621, KT022367, KX302407, KX302503	D	10
319	PK_JF804735-JF804737, JF804K5-JF804K8, KX232606, KX232618-KX232619, KY435515, KY435528, MH819267	D	13
	GQ431965, GQ429867	D	2
331	JF804694, KX232626, KY435526, MH819266, MH819269	R	5
372	JF804726, JF804735-JF804737, KX232594, KX232600, KX232620-KX232621, KY435528, KY435530-KY435532, KY435566, MH819273, MH819275	H	15
	AF184442, AF184458, AY492759, AY772986, DQ367290, FJ623491, GQ430209, GQ430357, GQ430393, GQ430447, GQ430479, GQ430564, GQ430825, GQ432033, GQ432183, GQ431950, GQ432307, GQ432337, KR020056, GQ432390, GQ432420, GQ432480, GQ432594, GQ432635, GQ432759, KR020074, KR020077, KR020105, KR781621, KR781626, KX234022, KX302407	H	33
373	AF184435, FJ623487, GQ429867, GQ430294, GQ431023, KR020087	PNGs	6
383	GU376767, GU376770, GU376772-GU376774, GU376776-GU376779, GU376781, GU376783-GU376785, GU376788-GU376792, JF804693-JF804709, JF804720-JF804726, JF804728-JF804737, JF804K0-JF804K9, KX232594-KX232595, KX232597, KX232600, KX232606-KX232607, KX232609-KX232611, KX232614, KX232618-KX232621, KX232624, KX232626, KY435514-KY435538, KY435541, KY435543, KY435564, KY435566, MH819263, MH819265-MH819267, MH819269, MH819272-MH819276, MH819279-MH819283	K	122
	AF184435, AF184440, AF184442, AF184453, AF184458, AF184462, AF457089, AF539405, AY492766, AY772986, DQ367289-DQ367290, FJ623487, FJ623491, GQ429867, GQ430035, GQ430209, GQ430294, GQ430357, GQ430447, GQ431023, GQ431950, GQ431965, GQ432033, GQ432183, GQ432249, GQ432390, GQ432420, GQ432480, GQ432635, GQ432759, KR020056, KR020074,KR020077, KR020087, KR020105, KR781621, KR781626, KT022367, KX234022, KX302407, KX302503, L11768	R	43
402	GU376792, JF804695, JF804703, JF804734, KX232594, KX232597, KX232609, KX232614, KY435519, KY435522, KY435525, KY435528-KY435529, KY435542, KY435566, MH819265-MH819266, MH819272, MH819274, MH819282-MH819283	K	21
	AY492766, KR020087	K	2

Figure S1. Genotyping of HIV-1 subtype A1.

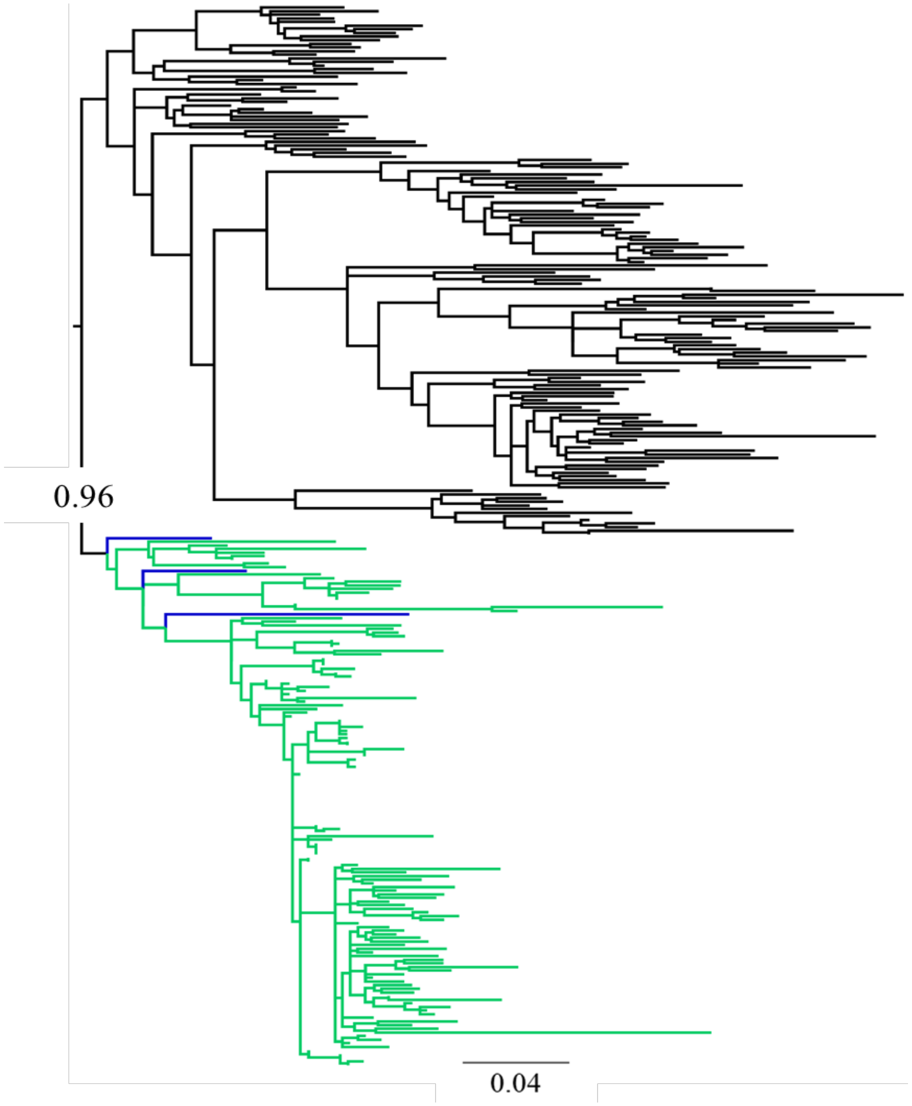
0.96
0.04

Figure S2. Clustering pattern of Kenyan reference dataset sequence with the all available Kenyan sequences from the Los Alamos HIV Database.

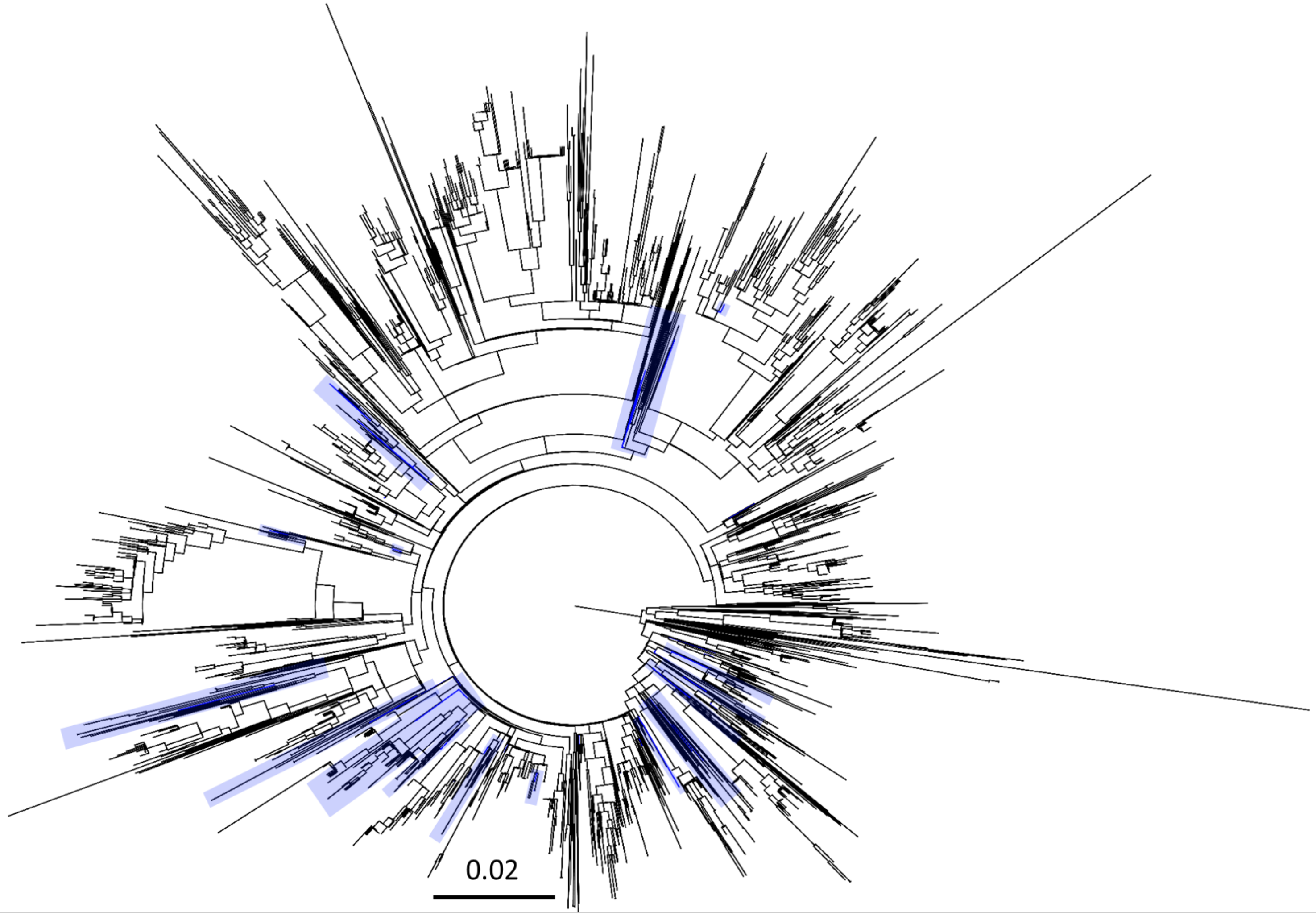

Figure S3. Amino Acid Variations within Pakistani and Kenyan cohort.

A.

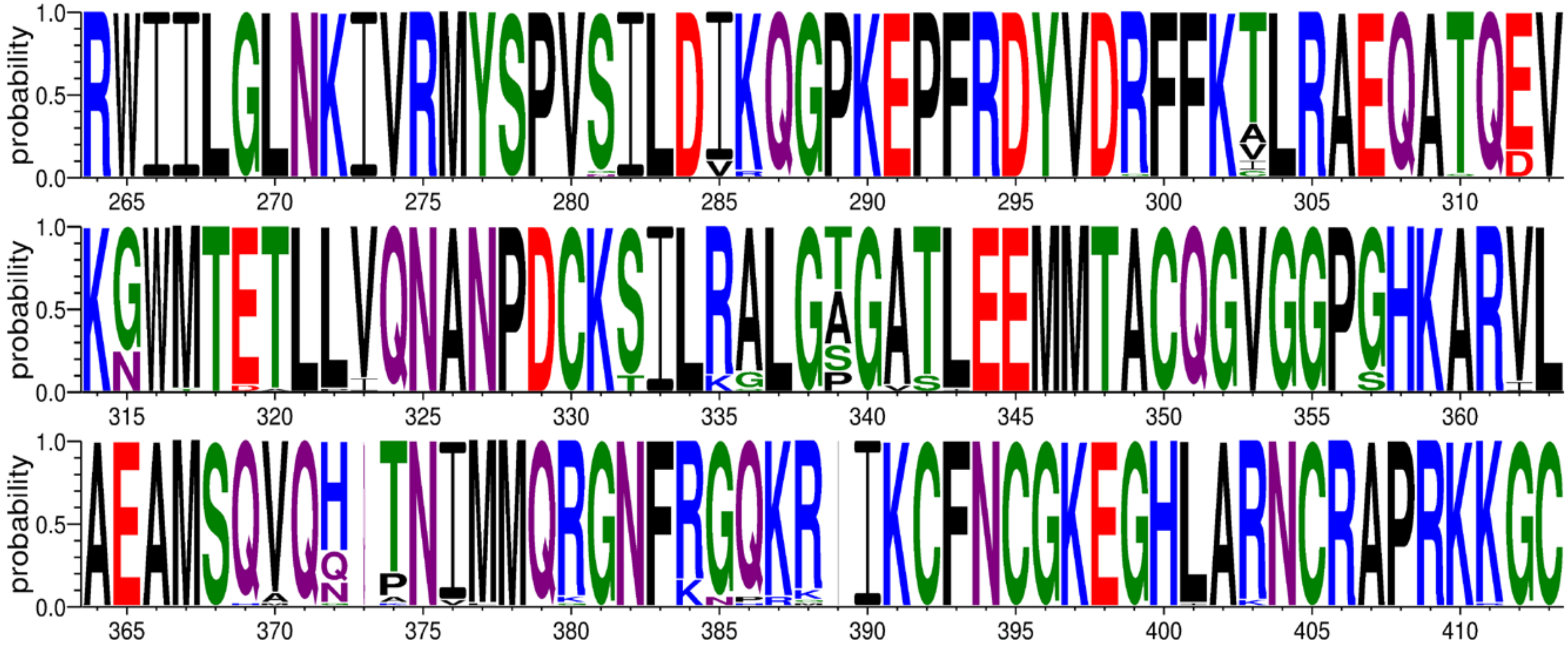

B.

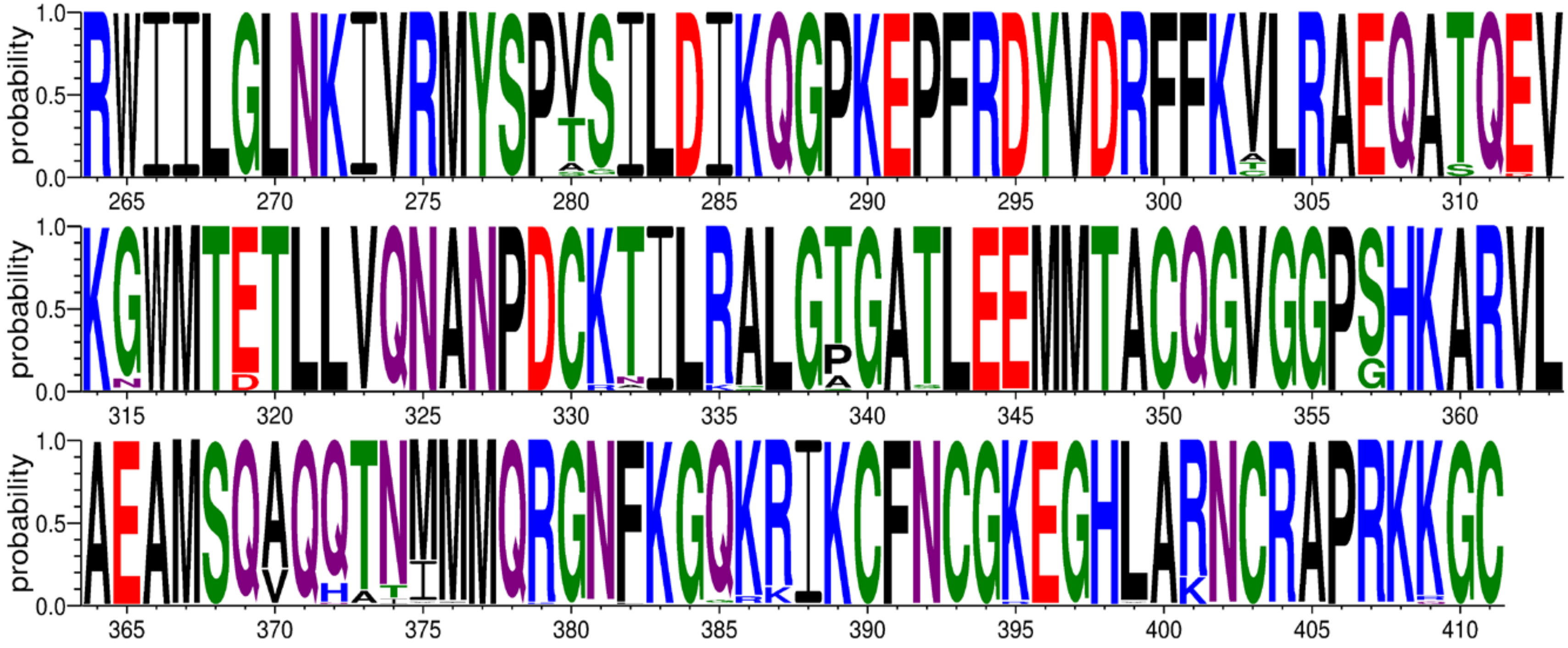
probability
1.0
0.5
0.0
265
270
275
280
285
290
295
300
305
310
315
320
325
330
335
340
345
350
355
360
365
370
375
380
385
390
395
400
405
410

Figure S4. Sites under selection pressure in HIV-1 subtype A1 Gag protein from Kenya and Pakistan.

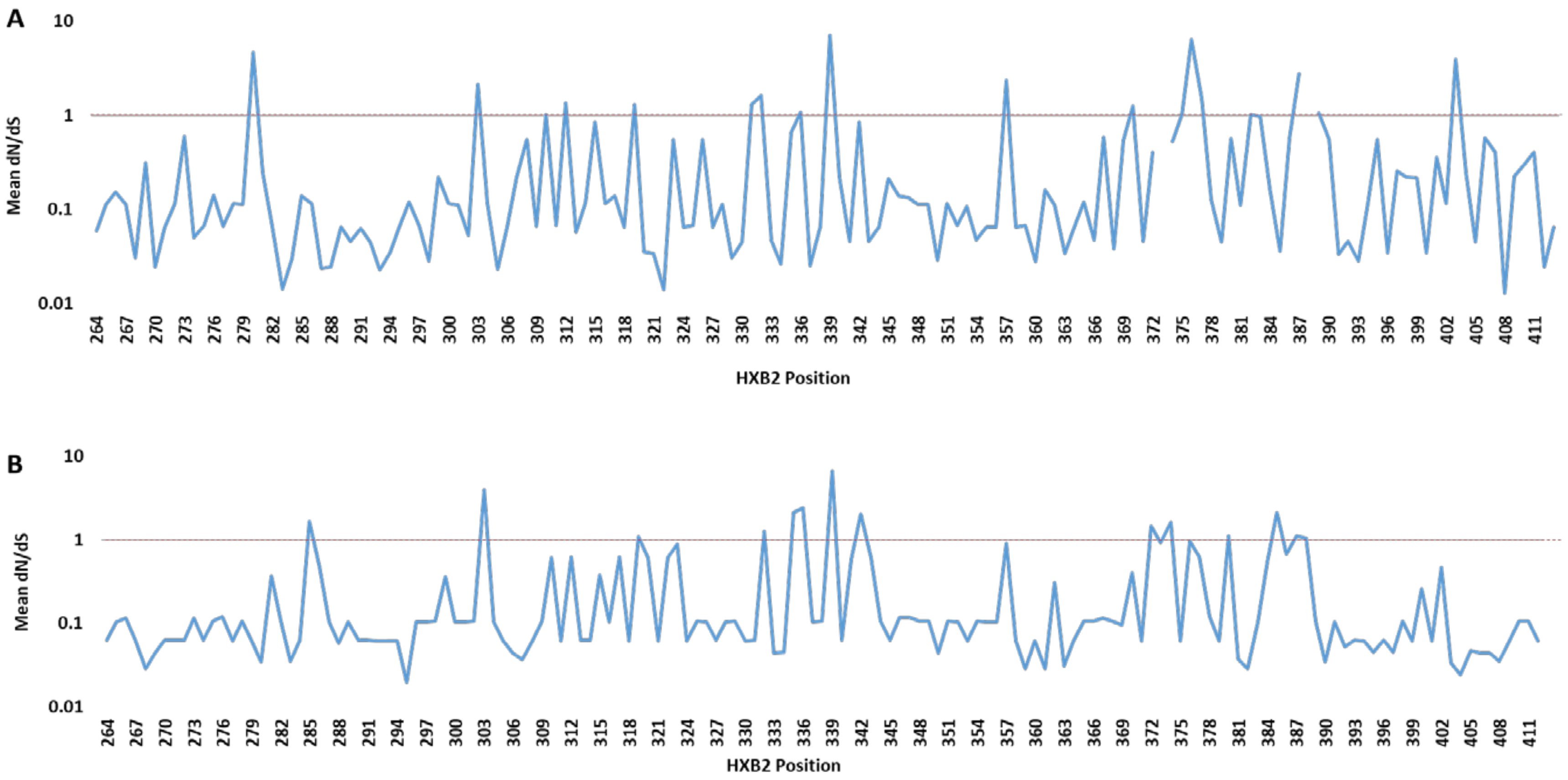

Supplement: Supplementary file 1 [file viruses-14-02307-s001.zip › viruses-1911418-supplementary.pdf]
